# Identification of cuproptosis -related subtypes, the development of a prognosis model, and characterization of tumor microenvironment infiltration in prostate cancer

**DOI:** 10.3389/fimmu.2022.974034

**Published:** 2022-09-20

**Authors:** Liang Jin, Wangli Mei, Xiang Liu, Xianchao Sun, Shiyong Xin, Zhen Zhou, Jiaxin Zhang, Bihui Zhang, Ping Chen, Ming Cai, Lin Ye

**Affiliations:** ^1^ Department of Urology, Shanghai East Hospital, School of Medicine, Tongji University, Shanghai, China; ^2^ Department of Urology, Shanghai Tenth People’s Hospital, School of Medicine, Tongji University, Shanghai, China; ^3^ Department of Orthopaedics, Shanghai Tenth People’s Hospital, School of Medicine, Tongji University, Shanghai, China; ^4^ Department of Urology, Shanghai Putuo District People’s Hospital, School of Medicine, Tongji University, Shanghai, China; ^5^ Department of Urology, The First Affiliated Hospital, and College of Clinical Medicine of Henan University of Science and Technology, Luoyang, China; ^6^ School of Life Sciences and Technology, Tongji University, Shanghai, China

**Keywords:** cuproptosis, prostate cancer, prognosis, immunotherapy, tumor immune microenvironment

## Abstract

Cuproptosis, Copper Induced Cell Death, is a newly defined type of programmed cell death, involving in the regulation of tricarboxylic acid (TCA) cycle. Dysfunction of cuproptosis induces cytotoxicity and influences the proliferation of multiple tumors. However, the direct prognostic effect of cuproptosis related genes and corresponding regulating mechanisms amid prostate cancer remains unknown. A multi-omics analysis strategy was adopted to explore the role of ten cuproptosis related genes in The Cancer Genome Atlas- Prostate Adenocarcinoma (TCGA-PRAD). Firstly, mRNA expression, Copy Number Variance (CNV), mutation, DNA methylation and prognostic power of the ten genes were illustrated. Based on transcriptomic data, we developed a novel prognostic model named the Cuproptosis-related gene score (CRGScore), Their biological functions were then detected by enrichment analysis and unsupervised cluster analysis. Following that, their correlation with Tumor Immune Microenvironment (TIME), immunotherapy, Biochemical Recurrence (BCR) and chemotherapeutic resistance were elaborated by relevant bioinformatics algorithms. Ten cuproptosis related genes exhibited extensive alteration of CNV and DNA methylation and showed significant influence on the prognosis of prostate cancer patients. These genes mainly enriched in E2F and G2M targets and mitosis pathways, Samples with high CRGScore showed enhancement resulting in the increased infiltration of T cell, B cell, NK cells. They also demonstrated close correlations with the BCR status, expression of eight immune checkpoints and chemotherapeutic resistances in prostate cancer. Our comprehensive analysis of CRGScore revealed an extensive regulatory mechanism by which they affect the tumor-immune-stromal microenvironment, clinicopathological features, and prognosis. We also determined the therapeutic liability of CRGScore in targeted therapy and immunotherapy. These findings highlight the crucial clinical implications of CRGScore and provide new ideas for guiding personalized immunotherapy strategies for patients with Pca.

## Introduction

Prostate cancer (PCa) is the second most common cancer in men and there are 191930 new diagnosed cases and 33330 deaths due to PCa in 2020 (1). Despite initial success with androgen-deprivation treatment, PCa patients gradually develop resistance after 1-2 years and progress to castration-resistant prostate cancer (CRPC), a condition that is incurable, ending up with inevitable death because of distal metastasis or tumor recurrence (2, 3). Hence, mining for novel biomarkers and molecular mechanisms for the prognosis and treatment of PCa is of great priority.

Cuproptosis (Copper Induced Cell Death) is a newly defined type of programmed cell death, differing from traditional cell death mechanism such as apoptosis, iron death and pyroptosis (4–7). Copper is a required cofactor for enzymes that mediate a host of essential cellular functions, including mitochondrial respiration, antioxidant defence and the biosynthesis of hormones, neurotransmitters and pigments (8), but at the same time dysregulation of copper stores can induce oxidative stress and cytotoxicity (9). Given the direct binding of copper to the lipoylated components of TCA cycle, dysfunction of cuproptosis can trigger cytotoxicity and influence the proliferation of tumor cells (10–12). In some research revealed cancer cells have a higher demand for copper compared with non-dividing cells (13), Copper imbalance can not only impact mitochondrial respiration but can also lead to changes in glycolysis, insulin resistance and lipid metabolism (14, 15). Beyond mitochondrial function, ATOX–ATP7A–LOX as copper pathways, could promote metastatic expansion. In addition, copper regulation of autophagy *via* ULK1 and ULK2 and/or protein quality control *via* UBE2D2 (16). However, the direct prognostic effect of cuproptosis related genes and corresponding regulating mechanisms amid prostate cancer cells remains unknown.

In this study, a multi-omics analysis strategy was adopted to explore the role of ten cuproptosis related genes in PRAD. Firstly, mRNA expression, CNV, mutation, DNA methylation and prognostic power of the ten genes were illustrated. Their biological functions were then detected by enrichment analysis and unsupervised cluster analysis. Following that, their correlation with TIME, immunotherapy, BCR and chemotherapeutic resistance were elaborated by relevant bioinformatics algorithms. The results of these analyses suggested that the cuproptosis related genes could influence the prognosis of PCa by involving in immune cell infiltration and mediating mitosis of cancer cells. These findings uncovered the role of cuproptosis and their underlying regulating mechanism in prostate cancer.

## Material and methods

### Multi-omics data source and obtain of ten cuproptosis related genes

Multi-omics datasets of prostate cancer were acquired from TCGA-PRAD cohort (496 tumor samples and 55 normal samples) at UCSC Xena website (17) (https://xenabrowser.net/datapages/). Detailed information of CNV, somatic mutation, DNA methylation (450k), RNA-seq in the format of TPM and survival data were retrieved for further analysis. 10 Cuproptosis related genes were collected, including DLAT, FDX1, MRF1, DLD, LIAS, LIPT1, PDHB, GLS, PDHA1, CDKN2A. In parallel, another cohort of prostate cancer from Gene Expression Omnibus (GEO) dataset (GSE54460) was downloaded for analysis to eliminate the heterogeneity of a single dataset.

### Differential analysis of ten cuproptosis related genes

R package limma (18) was utilized to seek out Cuproptosis related differentially expressed genes (DEGs) between 496 tumor and 55 normal samples of TCGA-PRAD cohort. R package ChAMP (19) was employed to identify differential methylation loci simultaneously. |log2 Fold Change (FC)| > 1 and False Discovery Rates (FDR) < 0.05 were set as the significant threshold for both analyses.

single nucleotide variants (SNV) and CNV information of the 10 Cuproptosis related genes were presented in the bubble and pie diagrams, it was acquired from GSCA website(http://bioinfo.life.hust.edu.cn/GSCA/#/drug). Meanwhile, correlation analyses of CNV and DNA methylation with mRNA value were conducted to reveal their impact on gene expression.

### Cluster analysis with ten cuproptosis related genes

To eliminate the heterogeneity of a single dataset, data of 496 tumor samples of TCGA-PRAD and 106 tumor samples of GSE54460 were merged and normalized by R function scale for further cluster analysis. Principal Component Analysis (PCA) depicted the heterogeneity before and after combination.

Next, unsupervised hierarchical clustering analysis was completed among the 602 tumor samples, with R package ConsensusClusterPlus (20), by setting the mRNA value of 10 Cuproptosis related genes as input information. PCA plot displayed the geometrical distance among sub-clusters. Differences of clinical information among sub-clusters were also illustrated in heatmap and boxplot.

### Identifying the functional difference and hub DEGs among three cuproptosis related sub-clusters

To elucidate the functional difference of the three Cuproptosis sub-clusters obtained from former cluster analysis, Gene Set Variation Analysis (GSVA) (21) were performed by using 50 Hallmarks-of-Cancer (22) and Kyoto Encyclopedia of Genes and Genomes (KEGG) (https://www.kegg.jp/) pathways as the background gene sets. Consequently, activities of these gene sets were quantified in each tumor sample. Next, heatmap was used to illustrated the differences of pathway activity between every two Cuproptosis related sub-clusters.

Furthermore, differential analysis was conducted between every two Cuproptosis related sub-clusters by using R package limma12. |log2 Fold Change (FC)| > 1 and FDR < 0.05 were set as the significant threshold. Volcano plot was used to display the DEGs of every two clusters. Following that, the intersected hub DEGs were recorded by using R function “intersect”, with Gene ontology (GO) (23) (http://wego.genomics.org.cn) and KEGG enrichment analysis depicting their biological function.

### Identifying the TIME difference among three cuproptosis sub-clusters

To identify the TIME difference among three Cuproptosis sub-clusters, Estimation of STromal and Immune cells in Malignant Tumours using Expression data (ESTIMATE) algorithm (24) was executed to computing the Stromal Score, Immune Score and ESTIMATE Score of each cluster. These scores reflected the stromal cells infiltration, immune cells infiltration and tumor purity respectively. Meanwhile, CIBERSORTx (25) was also carried out to calculate the infiltrating proportion of 22 types of immune cells among each cluster.

### Prognostic value of the hub DEGs among three cuproptosis sub-clusters

To test the prognostic value of the hub DEGs among three Cuproptosis sub-clusters, survival analysis and univariable Cox regression were implemented. Kaplan-Meier curve (K-M curve) illustrated patients’ survival difference by stratifying them into two groups, according to the median value of each hub DEG with significant prognostic power.

### Cluster analysis with seven hub DEGs

Whereafter, 7 hub DEGs with significant prognostic power were subjected to the second time unsupervised cluster analysis, resulting in two GeneCluster. K-M curve and heatmap were then depicted to demonstrated the survival and clinic pathological differences of the GeneCluster.

### Prognostic value of the genecluster

To check the robustness of the GeneCluster, PCA was performed to show the geometrical distance and patients were stratified into two groups according to the median PCA score. Afterwards, consistency of the two PCA group and the two sub-clusters in GeneCluster were estimated in the Sankey diagram. Survival difference of the two PCA group and correlation of PCA score with immune cell infiltration was then analyzed after computing the infiltrating proportion of 22 types of immune cells.

### Correlation with biochemical recurrence and immunotherapy

To explore the correlation of PCA score with BCR of prostate cancer, BCR status of patients in two PCA groups were presented in the boxplot. Then, expression difference of six immune checkpoints in two PCA groups was also detected to test its correlation with immunotherapy. Following that, correlation of PCA score with drug resistance was also discovered by computing the IC50 of twelve typical chemotherapeutics with R package pRRophetic (26).

### Statistic and software

Data processing and analysis were accomplished by R 4.0.4. [Package: limma, ggplot2, survminer, ChAMP, ggcorrplot, GSVA, CIBERSORTx and so on (21, 27, 28)]. Student T test and ANOVA test were applied for comparisons between groups, while Pearson correlation were adopted to estimate the statistical correlation of parametric variables. Two-sided P < 0.05 was considered as significant threshold for all statistical tests.

## Results

### Differential expression, SNV, CNV and DNA methylation of ten cuproptosis related genes in prostate cancer

**Figure 1:**
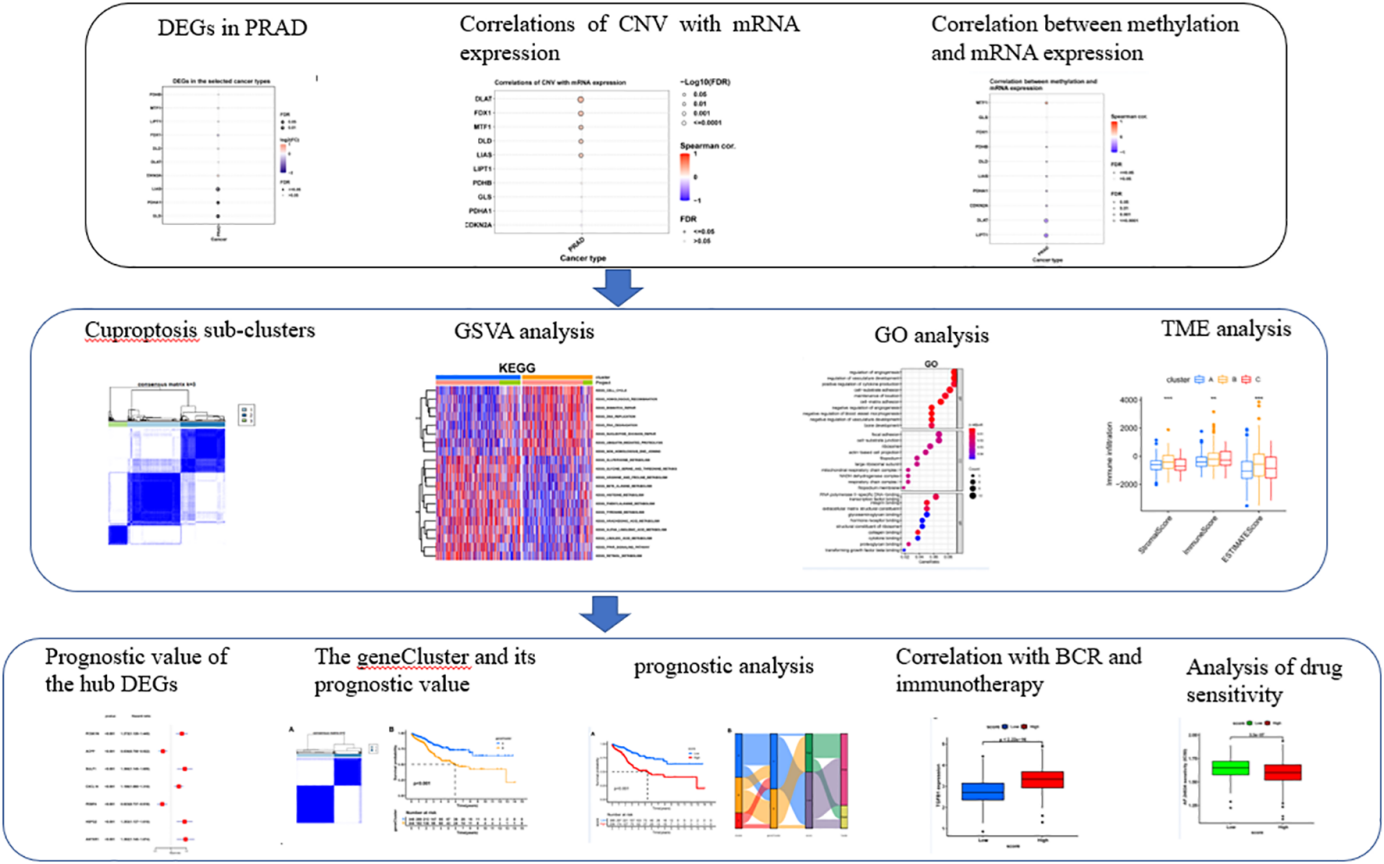
The schematic diagram of analysis.

**Figure 2:**
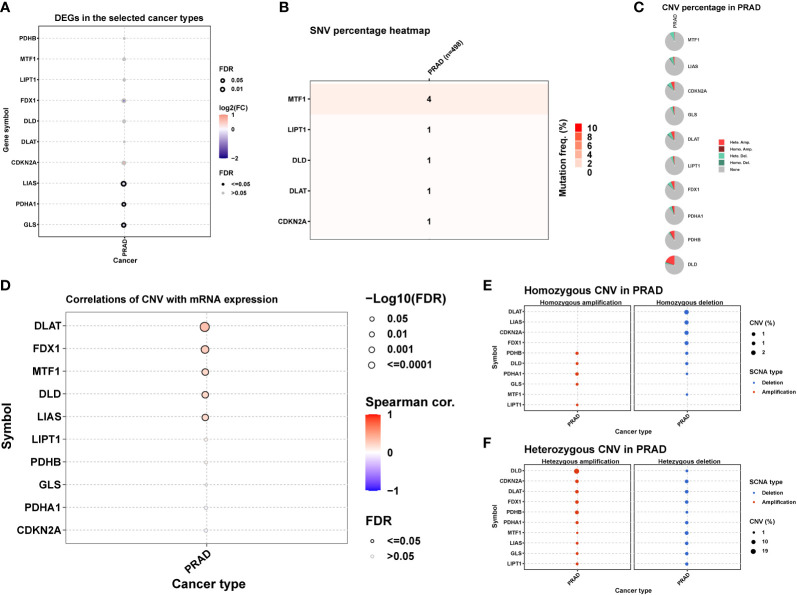
Differential expression, SNV and CNV of ten Cuproptosis related genes in prostate cancer. **(A)** Differential expression of Cuproptosis related genes in prostate cancer. **(B)** The frequency of deleterious mutations. **(C)** Pie plot summarizes the CNV of genes in indicated tumor types. **(D)** The correlation between CNV with gene expression. **(E)** Figure provides the profile of homozygous CNV of genes in PRAD. **(F)** Figure provides the profile of heterozygous CNV of genes in PRAD.

**Figure 1** was showing the schematic diagram of analysis. Of the ten Cuproptosis related genes, three were found to be significantly down-regulated in prostate cancer (**Figure 2A**). MTF1 was the most frequently mutant gene (**Figure 2B**) while DLD had the highest level of heterozygous copy number amplification (**Figures 2C, E, F**).

**Figure 3:**
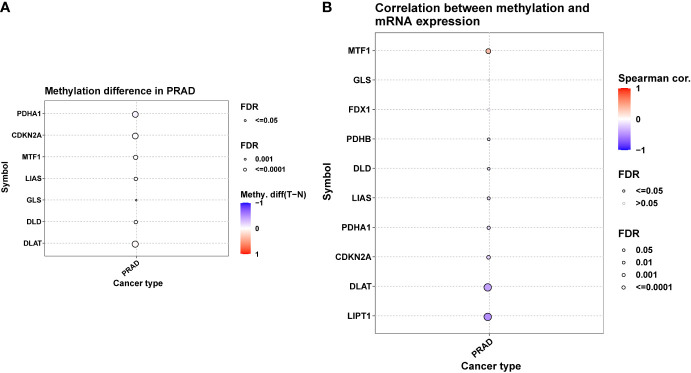
DNA methylation of ten Cuproptosis related genes in PRAD. **(A)** Figure summarizes the methylation difference between tumor and normal samples of in PRAD. **(B)** The correlation between methylation and mRNA expression of each gene.

A positive correlation between CNV and mRNA expression was seen in DLAT, FDX1, MTF1 i.e., (**Figure 2D**). DNA methylation, however, induced extensive down-regulation of their mRNA expression (**Figures 3A, B**). Furthermore, Correlation between expression and immune infiltrates in PRAD was shown in **Figure S1**.

### Three cuproptosis sub-clusters and their functional differences

**Figure 4:**
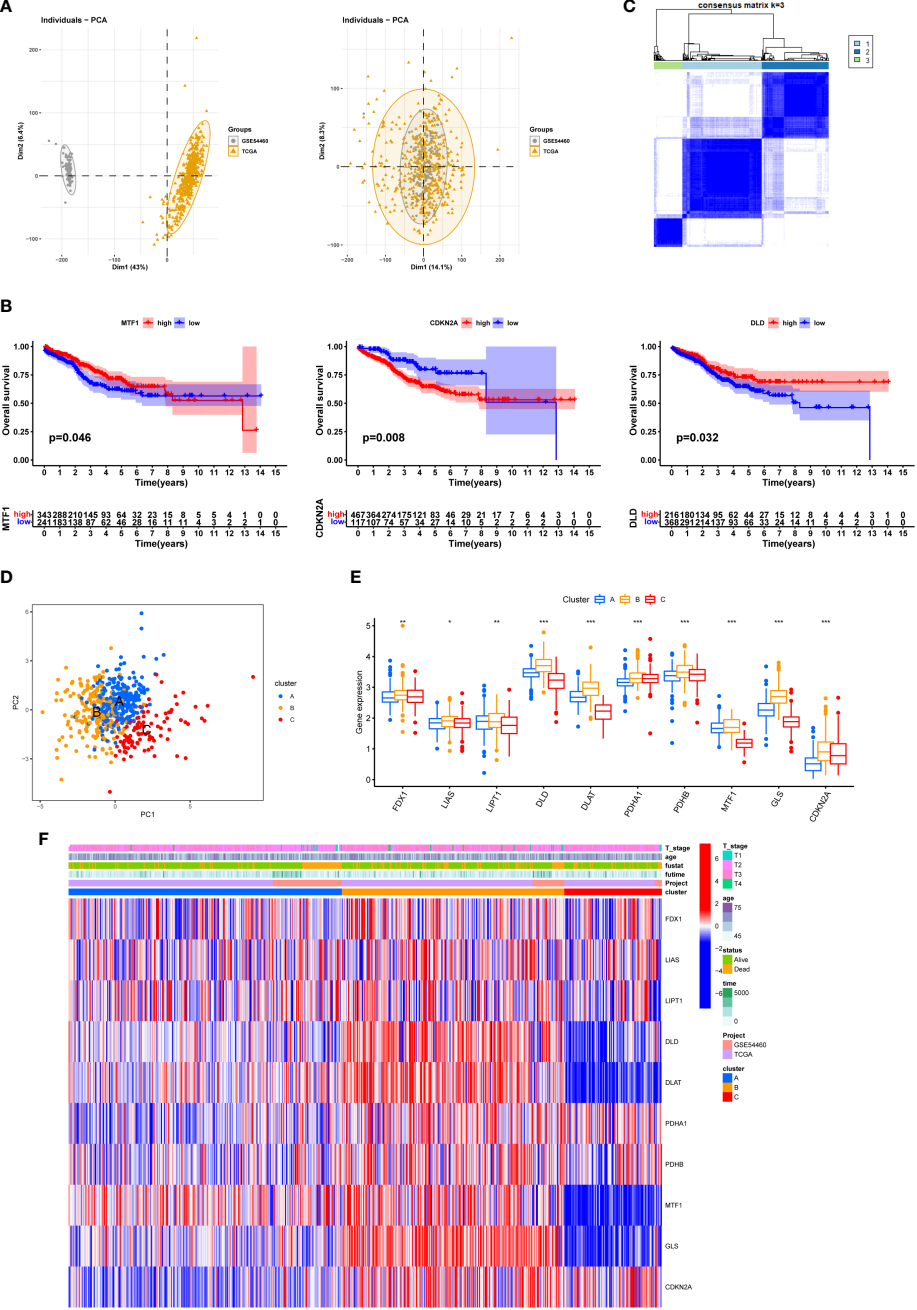
Three Cuproptosis sub-clusters were performed. **(A)** Use PRAD in TCGA and GSE54460 to merge data and use R package “limma” and “sva” to remove batch effects. **(B)** KM survival curve for each Cuproptosis related genes. **(C)** Unsupervised clustering analysis. **(D)** PCA diagram showing the distribution of different sub-clusters. **(E)** Differential expression of Cuproptosis related genes between different sub-clusters. **(F)** heatmap showing the relationship between clinical features, genes expression and sub-clusters. * represents p<0.05,** represents p<0.01,***represents p<0.001, ns represents p>0.05.

Different datasets were separated in the PCA plot because of heterogeneity which was eliminated after normalization (**Figure 4A**). Three of ten Cuproptosis related genes showed a significant influence on patients’ survival outcome in the combined data (**Figure 4B**). The merged dataset was then divided into three categories by using the expression of ten Cuproptosis related genes (**Figure 4C**). The PCA plot showed the geometrical distance and different gene expression patterns were seen among the three sub-clusters (**Figures 4D, E**). A general high expression of Cuproptosis related genes was observed in Cluster B and patients in cluster B seemed to have a better survival outcome when compared with cluster A and cluster C (**Figures 4E, F**).

**Figure 5:**
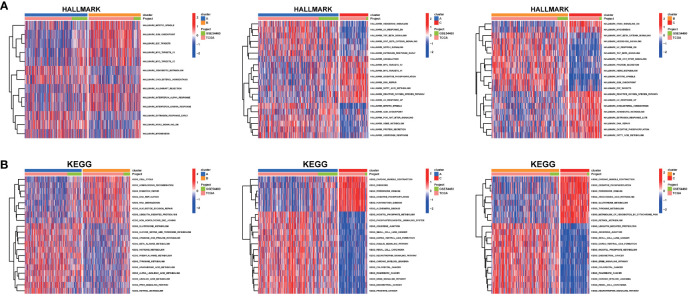
GSVA analysis. The **(A)** HALLMARK pathway, **(B)** KEGG pathway were downloaded separately from the Msigdb database and the pathways were scored using the R package GSVA.

There were huge functional differences among the three sub-clusters. Hallmark activities of E2F TARGETS, G2M CHECKPOINT and MITOTIC SPINDLE were consistently higher in cluster B than cluster A and C (**Figures 5A-C**). Tumor in cluster B also possessed a more active function in CELL CYCLE and MISMATCH REPAIR than cluster A, as well as increased activity of Ubiquitin Mediated Proteolysis pathway than cluster C (**Figures 5D-F**).

**Figure 6:**
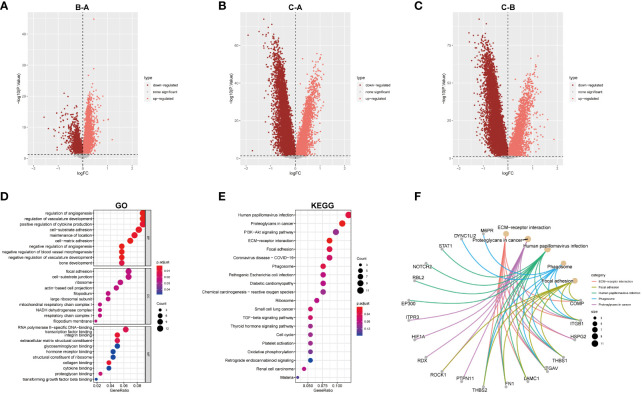
GO and KEGG analysis. **(A–C)** Differential analysis of the three subtypes. **(D)** GO analysis was conducted and visualized. **(E)** KEGG analysis was conducted and visualized. **(F)** the correction between genes and pathways in top5 KEGG results.

DEGs among the three sub-clusters were demonstrated in the volcano plots (**Figures 6A-C**). 180 hub DEGs were acquired after taking their intersection by R function “intersect”. Further Go and KEGG analysis found the 180 hub DEGs were mainly enriched in angiogenesis, focal adhesion, proteoglycans pathways and so on (**Figures 6D-F**).

### Different TME pattern among the three cuproptosis sub-clusters

**Figure 7:**
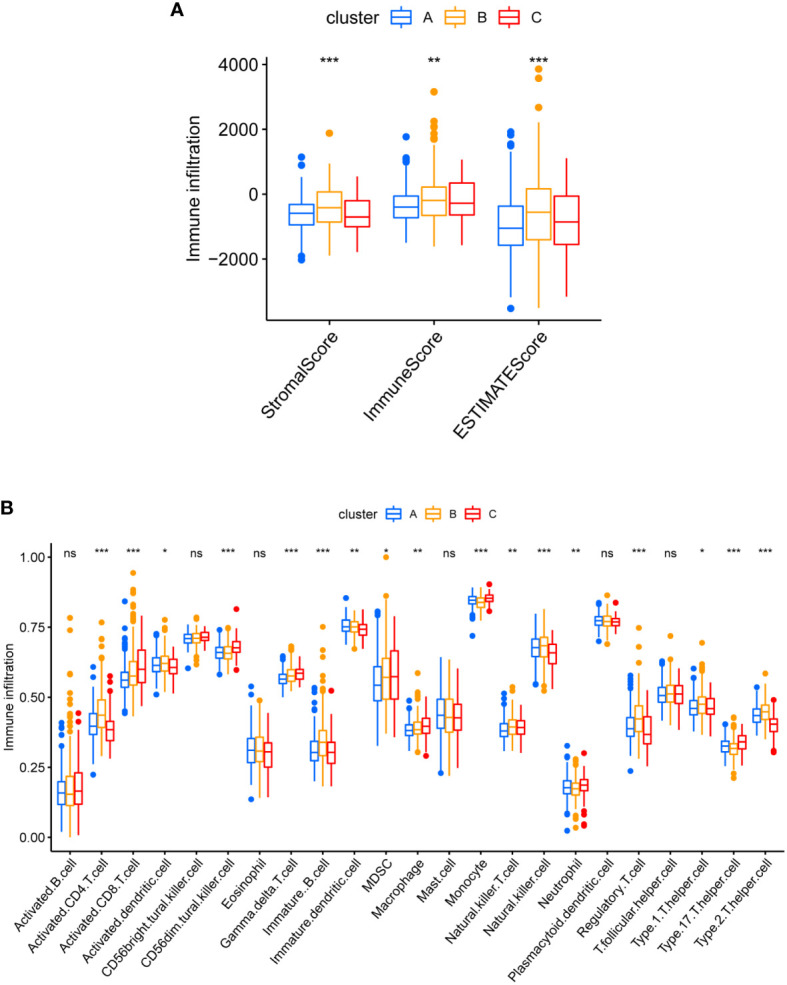
Different TME pattern among the three Cuproptosis sub-clusters. **(A)** Differences between Stromal Score, Immune Score and ESTIMATE Score in different typologies. **(B)** Differences in immune cell infiltration between different subtypes. * represents p<0.05, ** represents p<0.01, *** represents p<0.001, ns represents p>0.05.

After computing the Stromal Score, Immune Score and ESTIMATE Score of each cluster by ESTIMATE algorithm, cluster B seemed to have the highest Immune Score and ESTIMATES core (**Figure 7A**). That suggested a higher proportion of immune cell in cluster B, keeping consistence with the result of CIBERSORTx where cluster B demonstrated more infiltration of CD4 T cell, B cell, NK cell and regulatory T cell (**Figure 7B**).

### Prognostic value of the hub DEGs among three cuproptosis sub-clusters

**Figure 8:**
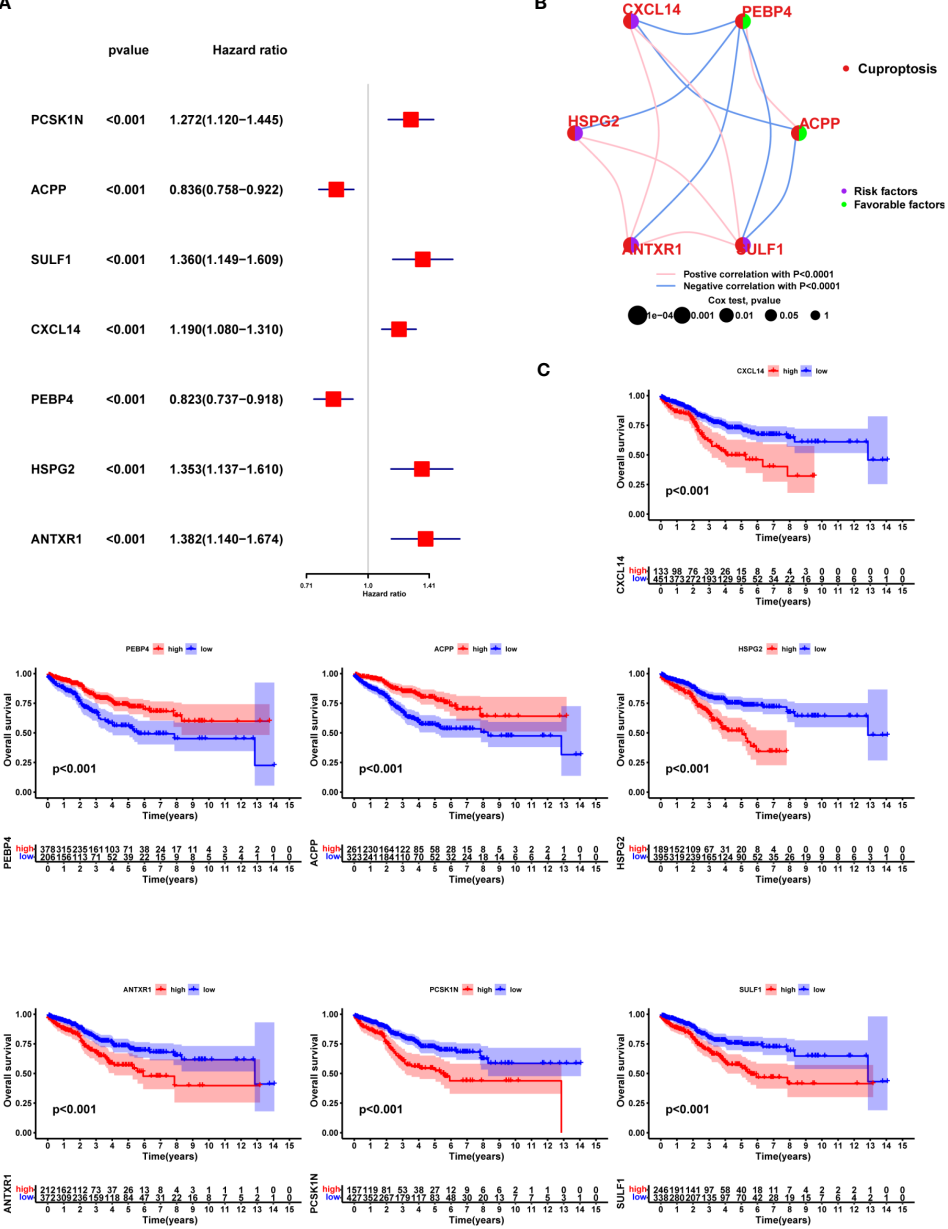
Prognostic value of the hub DEGs among three Cuproptosis sub-clusters. **(A)** Seven of one hundred and eighty hub DEGs among the three Cuproptosis sub-clusters demonstrated noticeable prognostic power in Cox regression. **(B)** correlation was observed among the seven hub DEGs **(C)** KM survival curve.

Seven of one hundred and eighty hub DEGs among the three Cuproptosis sub-clusters demonstrated noticeable prognostic power in Cox regression (**Figure 8A**). Close correlation was observed among the seven hub DEGs (**Figure 8B**). Of them, PEBP4 and ACPP seemed to be protective factors with a HR of 0.823 (0.737-0.918) and 0.836 (0.758-0.922) (**Figure 8C**). The univariable Cox regression analysis was shown in **Table S1**.

### The genecluster and its prognostic value

**Figure 9:**
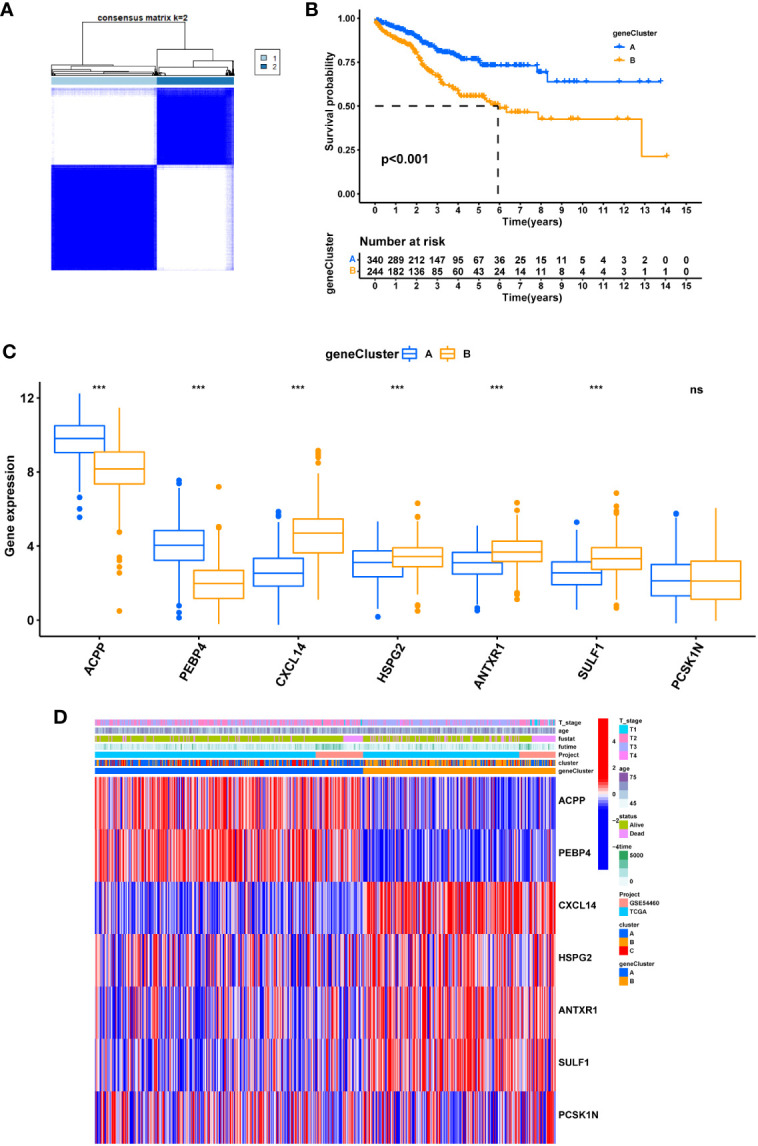
The geneCluster and its prognostic value. **(A)** sub-clusters were performed with differential genes. **(B)** Survival analysis in PRAD. **(C)** Differential expression of Cuproptosis related genes between geneCluster. **(D)** heatmap showing the relationship between clinical features, genes expression and sub-clusters. * represents p<0.05, ** represents p<0.01, *** represents p<0.001, ns represents p>0.05.

Two clusters were then obtained in unsupervised clustering by setting the expression of seven hub DEGs as input information (**Figure 9A**). The geneCluster well stratified patients into two groups with considerable difference in survival outcome (**Figure 9B**) and gene expression pattern (**Figures 9C, D**).

**Figure 10:**
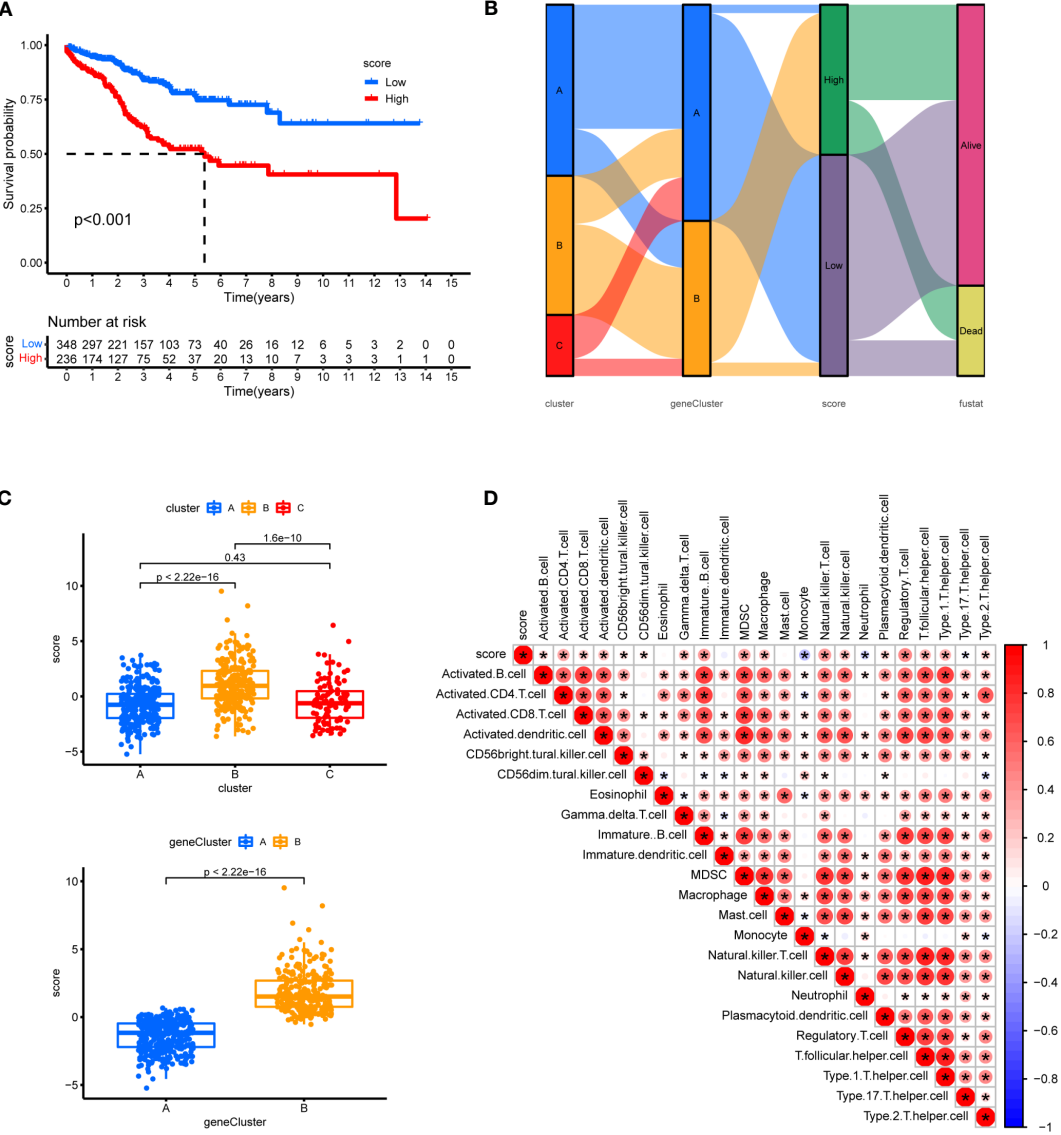
Prognostic analysis. **(A)** Using PCA analysis, scores were calculated based on 7 genes, survival analysis between high and low scores was shown. **(B)** Sankey diagram showing the relationship between staging, scoring and prognostic status. **(C)** Differences in geneCluster scores for Cuproptosis sub-clusters and Differences cluster scores for 7 hub genes. **(D)** Correlation of immune cell infiltration. One asterisk (*) indicates p value smaller than 0.05 (P< 0.05). Size and color of the circle represent the Pearson correlation coefficients. * indicates p value smaller than 0.05 (P< 0.05).

To check the robustness of the GeneCluster, PCA was performed where the PCA score also divided patients into two groups with significant survival difference (**Figure 10A**). In the Sankey diagram, rather good consistency was seen for two gene clusters to two PCA groups (**Figure 10B**). The PCA score also differs in the three Cuproptosis sub-clusters (**Figure 10C**). Moreover, the PCA score was positively correlated with the majority of infiltrating immune cells except for monocyte (**Figure 10D**).

### Correlation with BCR, immunotherapy and chemotherapeutics resistance

**Figure 11:**
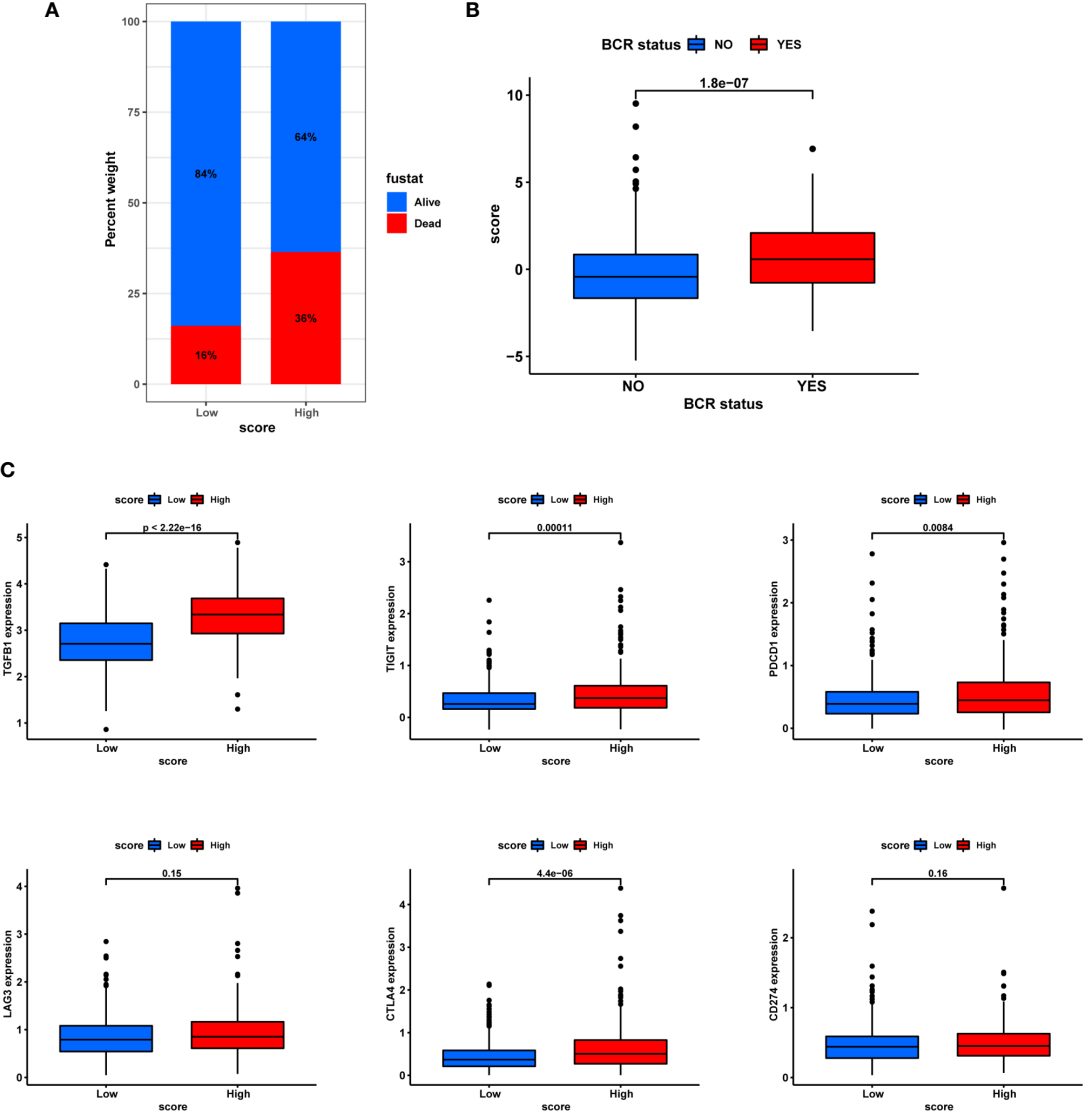
Correlation with BCR and immunotherapy. **(A)** Ratio of BCR status and score. **(B)** Differences in BCR status and score. **(C)** Differential expression of immune checkpoints in different subgroups.

In terms of clinic pathological features, the PCA score also showed certain ability to distinguish patients’ survival and BCR status (**Figures 11A, B**). In addition, the T stage status was shown in **Figure S2**. Then, in line with the extensive positive correlation of PCA score with most infiltrating immune cells, there was increased expression of eight immune checkpoints in the groups with higher PCA score (**Figure 11C**). These results suggested a higher tendency for patients in the high-score group to be responsive to immunotherapy.

**Figure 12:**
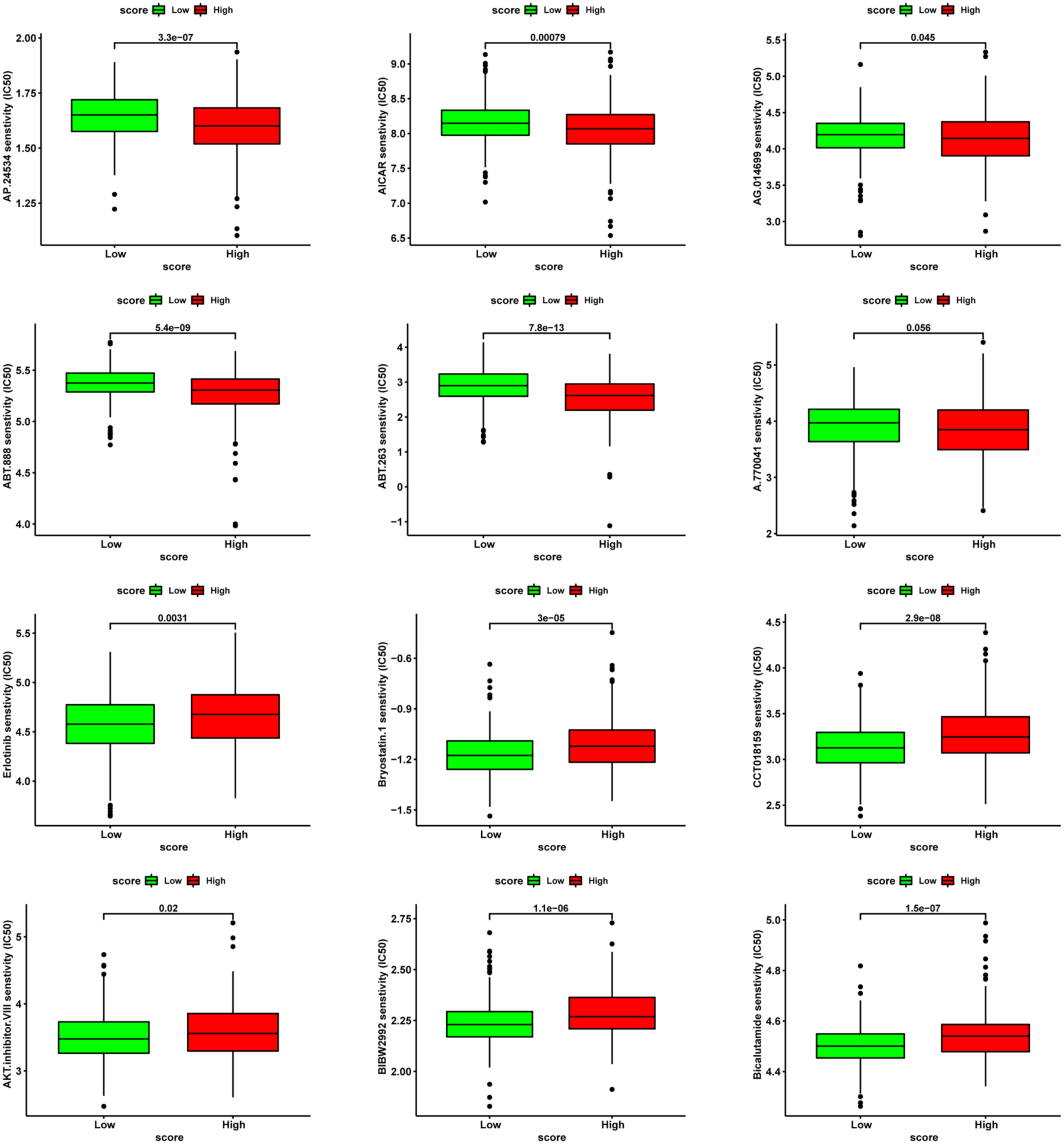
Analysis of drug sensitivity associated with CRGScore. Predicting IC50 values for multiple anti-cancer drugs.

With regards to chemotherapy, patients in the high-score group were less likely to benefit from Bicalutamide and AKT inhibitor VIII but more likely to benefit from multitarget kinase inhibitors Ponatinib (AP24534), Bcl-2 inhibitor ABT.263 and PARP1 inhibitor ABT.888 (**Figure 12**).

## Discussion

In this study, multidimensional analyses were implemented to explore the biological function, prognostic ability, correlation with TIME, immunotherapy, BCR (Biochemical Recurrence) and chemotherapeutic resistance of ten cuproptosis related genes in PRAD The result suggested that cuproptosis could influence the prognosis of PCa by involving in immune cell infiltration and mediating mitosis of cancer cells. To our knowledge, this is the first study to elucidate the role of cuproptosis and its potential regulating mechanism in prostate cancer.

Surprisingly, seven of ten genes showed no expression difference between tumor and control samples. However, this does not weaken their importance as the alteration of SNV, CNV or methylation they possessed are equally critical in prostate cancer pathogenesis. Taking CDKN2A (Cyclin-Dependent Kinase Inhibitor 2A) for example, it ranked high in the list of homozygous deletion and methylation difference and was a significant prognostic risk factor for PCa. Commonly, CDKN2A was a well-known tumor suppressor which can generates 14 alternative transcripts, the best known being p16(INK4a), to induce cell G2 arrest and apoptosis in a p53-independent manner (29). Thus, mutation and homozygous deletion of CDKN2A make it an adverse prognostic factor in a variety of cancers (30–33), which is in line with our result.

With regards to the biological function, cluster B demonstrated increased activity of MITOTIC SPINDLE, more infiltration of CD4 T cell, B cell, NK cell and regulatory T cell with noticeable higher expression of DLD, DLAT, MTF1 and GLS than A and C. Dihydrolipoamide S-Acetyltransferase (DLAT) was one of the components of the pyruvate dehydrogenase (PDH) complex (34) and involved in glycolysis and mitochondrial respiratory function of substantial cell types including immune T cell (35). In parallel, DLAT expression was also found to be positively correlated with immune B cell infiltration and CD274 expression in clear cell renal cell carcinoma (12). These alike evidences suggested a favourable role of DLAT to promote antigen presenting and immune response.

Our study has several advantages. Above all, this is the first study to elucidate the role of cuproptosis and its potential regulating mechanism in prostate cancer from multi dimensions. Moreover, as cell death is fundamental to cancer origin (36) and intracellular copper within a certain range has exhibited a selectively killing tendency towards tumor cells (9), our findings may bring us better therapeutic targets for the treatment of prostate cancer. In addition, we employed combined analysis of two datasets to eliminated the heterogeneity from single repository as much as possible and the extra PCA analysis confirmed the robustness of the unsupervised clustering.

There were also several limitations in our study. First, external validation for the expression and prognostic ability of relevant genes would make the results more convincing. Second, the inherent fault of data-mining from public databases is inevitable, further *in vitro* or *in vivo* biological evidences are needed.

Our comprehensive analysis of CRGScore revealed an extensive regulatory mechanism by which they affect the tumor-immune-stromal microenvironment, clinicopathological features, and prognosis. We also determined the therapeutic liability of CRGScore in targeted therapy and immunotherapy. These findings highlight the crucial clinical implications of CRGScore and provide new ideas for guiding personalized immunotherapy strategies for patients with Pca.

## Data availability statement

The datasets presented in this study can be found in online repositories. The names of the repository/repositories and accession number(s) can be found in the article/**Supplementary Material**.

## Author contributions

PC, MC, and LY conceived and designed experiments, and they contributed equally to this work. LJ, WM, XL, XS, SX, ZZ, JZ, and BZ conducted the experiments and obtained the results. LJ, WM, and XL, sorted and analyzed the results. LJ, and WM, wrote the draft. PC, MC, and LY. extensively revised, formatted, and submitted versions of the manuscript. All authors participated in data discussions and have seen and approved the submitted version of manuscript.

## Funding

This work was supported by grants from the National Natural Science Foundation of China (No.81972409, 81672549), Health System Independent Innovation Science Foundation of Shanghai Putuo District (No.PTKWWS201819).

## Conflict of interest

The authors declare that the research was conducted in the absence of any commercial or financial relationships that could be construed as a potential conflict of interest.

## Publisher’s note

All claims expressed in this article are solely those of the authors and do not necessarily represent those of their affiliated organizations, or those of the publisher, the editors and the reviewers. Any product that may be evaluated in this article, or claim that may be made by its manufacturer, is not guaranteed or endorsed by the publisher.
