# Supplementary material for: Identification of cuproptosis -related subtypes, the development of a prognosis model, and characterization of tumor microenvironment infiltration in prostate cancer
Source: Front Immunol. 2022 Sep 20;13:974034. doi: 10.3389/fimmu.2022.974034 (PMC9530990; doi:10.3389/fimmu.2022.974034)
Supplement: Supplementary file 1 [file DataSheet_1.zip › supplementary materials/Figure legends.docx]

**Figure legends**

**Figure S1**：Correlation between expression and immune infiltrates in PRAD

**Figure S2**：Correlation with T stage in different score. (A) Ratio of T stage and score. (B) Differences in T stage and score.
