# Supplementary material for: Identification of cuproptosis -related subtypes, the development of a prognosis model, and characterization of tumor microenvironment infiltration in prostate cancer
Source: Front Immunol. 2022 Sep 20;13:974034. doi: 10.3389/fimmu.2022.974034 (PMC9530990; doi:10.3389/fimmu.2022.974034)
Supplement: Supplementary file 1 [file DataSheet_1.zip › supplementary materials/Table S1.docx]

Table 1: Univariate Cox regression analysis in prostate cancer

| Id | HR | HR.95L | HR.95H | *P* value |
| --- | --- | --- | --- | --- |
| ACPP | 0.83612 | 0.757994 | 0.922299 | 0.000349 |
| PEBP4 | 0.822653 | 0.73728 | 0.917912 | 0.000479 |
| ANPEP | 0.943737 | 0.881163 | 1.010755 | 0.098058 |
| COMP | 1.380365 | 1.242004 | 1.53414 | 2.21E-09 |
| CXCL14 | 1.189508 | 1.08027 | 1.309793 | 0.000414 |
| RLN1 | 0.992524 | 0.927641 | 1.061945 | 0.827789 |
| TFF3 | 0.9872 | 0.914904 | 1.065209 | 0.739898 |
| MSMB | 0.931143 | 0.862213 | 1.005585 | 0.06906 |
| ALOX15B | 0.950526 | 0.88821 | 1.017214 | 0.142479 |
| CRISP3 | 1.055097 | 0.995576 | 1.118176 | 0.070248 |
| NPY | 0.942788 | 0.896439 | 0.991533 | 0.02199 |
| DYNC1LI2 | 0.834975 | 0.678559 | 1.027446 | 0.088358 |
| SMARCC2 | 1.063219 | 0.820942 | 1.376997 | 0.642218 |
| SEC24C | 1.184633 | 0.960899 | 1.460462 | 0.112629 |
| EP300 | 1.189385 | 0.904396 | 1.564179 | 0.21462 |
| USP34 | 0.832719 | 0.666372 | 1.040591 | 0.107393 |
| ZNF770 | 0.858159 | 0.704476 | 1.045369 | 0.128692 |
| EHF | 0.907295 | 0.788697 | 1.043728 | 0.173463 |
| RBL2 | 0.957625 | 0.764038 | 1.200261 | 0.707082 |
| NT5C2 | 0.913845 | 0.737138 | 1.132912 | 0.411221 |
| PRRC2B | 1.44986 | 1.148641 | 1.83007 | 0.001771 |
| GTF2I | 1.07999 | 0.85954 | 1.35698 | 0.508863 |
| ITGB1 | 0.887421 | 0.735875 | 1.070176 | 0.211269 |
| DENND4C | 0.787407 | 0.625971 | 0.990476 | 0.04118 |
| M6PR | 0.954687 | 0.74758 | 1.219171 | 0.710146 |
| NDUFA13 | 1.140182 | 0.910456 | 1.427873 | 0.253129 |
| BCL9L | 1.454306 | 1.108576 | 1.907859 | 0.006847 |
| NFAT5 | 0.718875 | 0.56167 | 0.920081 | 0.008754 |
| NDUFB7 | 1.116196 | 0.869526 | 1.432843 | 0.388287 |
| TBC1D14 | 1.357184 | 1.068421 | 1.723992 | 0.012343 |
| GADD45GIP1 | 1.219599 | 0.957578 | 1.553317 | 0.107682 |
| ATP5D | 1.08938 | 0.877078 | 1.35307 | 0.438899 |
| LPCAT3 | 0.953406 | 0.775646 | 1.171904 | 0.65039 |
| DLG5 | 1.124484 | 0.900358 | 1.404401 | 0.300913 |
| PCDHGC3 | 0.92534 | 0.737237 | 1.161436 | 0.503355 |
| C16orf13 | 1.318425 | 1.050616 | 1.654499 | 0.017024 |
| ROMO1 | 1.341714 | 1.101322 | 1.634578 | 0.003522 |
| RPLP2 | 1.164514 | 0.932605 | 1.454092 | 0.178892 |
| TMEM160 | 1.305474 | 1.048255 | 1.625809 | 0.017271 |
| RAB11FIP1 | 0.940964 | 0.759553 | 1.165704 | 0.57763 |
| RPL36A | 0.986105 | 0.786773 | 1.235939 | 0.903341 |
| RAB23 | 1.040327 | 0.833567 | 1.298372 | 0.726556 |
| ACER2 | 0.943746 | 0.760793 | 1.170694 | 0.598474 |
| UTRN | 0.971649 | 0.77697 | 1.215106 | 0.800953 |
| DSG2 | 0.844395 | 0.686027 | 1.039322 | 0.110485 |
| NME3 | 1.351099 | 1.089476 | 1.675547 | 0.006137 |
| FAT1 | 1.099703 | 0.915149 | 1.321474 | 0.310596 |
| ADAM9 | 0.956315 | 0.794772 | 1.150694 | 0.636111 |
| HSPG2 | 1.353057 | 1.137463 | 1.609515 | 0.000639 |
| CCDC85B | 1.248402 | 1.053534 | 1.479313 | 0.0104 |
| C4orf48 | 1.195661 | 1.012988 | 1.411275 | 0.034642 |
| TMSB10 | 1.456878 | 1.213252 | 1.749426 | 5.57E-05 |
| TMEM238 | 1.189375 | 0.992895 | 1.424735 | 0.05976 |
| SEPP1 | 0.917823 | 0.776881 | 1.084336 | 0.313411 |
| BHLHA15 | 1.076692 | 0.931884 | 1.244002 | 0.316011 |
| RPL21 | 1.16501 | 1.012452 | 1.340555 | 0.032943 |
| THBS1 | 0.973769 | 0.85528 | 1.108673 | 0.688022 |
| FBXO11 | 0.858465 | 0.65006 | 1.133684 | 0.2821 |
| MFAP3 | 0.96365 | 0.74133 | 1.252644 | 0.782019 |
| HEATR1 | 0.970153 | 0.708255 | 1.328896 | 0.850292 |
| NR2C2 | 1.082635 | 0.792341 | 1.479287 | 0.618121 |
| PTPN11 | 0.84142 | 0.628694 | 1.126123 | 0.245575 |
| RASA1 | 0.804413 | 0.611831 | 1.057613 | 0.119048 |
| ROCK1 | 1.142983 | 0.84735 | 1.541761 | 0.381465 |
| STAG1 | 1.229499 | 0.906705 | 1.667209 | 0.183629 |
| NUP153 | 0.823141 | 0.613331 | 1.104724 | 0.194801 |
| RECQL | 1.147288 | 0.847897 | 1.552393 | 0.373168 |
| PIK3C2A | 0.727881 | 0.556709 | 0.951684 | 0.020233 |
| VGLL4 | 1.230018 | 0.914284 | 1.654786 | 0.171352 |
| FAM208B | 1.105538 | 0.818037 | 1.494081 | 0.513806 |
| TSTD2 | 0.796452 | 0.598668 | 1.059578 | 0.118141 |
| IPO7 | 0.878842 | 0.655492 | 1.178296 | 0.387985 |
| TAF2 | 1.400412 | 1.037567 | 1.890147 | 0.027737 |
| SPTLC1 | 0.952078 | 0.725756 | 1.248978 | 0.72289 |
| ASAP2 | 1.231654 | 0.919971 | 1.648934 | 0.161621 |
| ASH1L | 0.917587 | 0.70571 | 1.193077 | 0.520826 |
| C5orf24 | 0.913057 | 0.690244 | 1.207794 | 0.523961 |
| RDX | 0.748896 | 0.569339 | 0.985081 | 0.038693 |
| EIF3A | 0.99514 | 0.755926 | 1.310054 | 0.972297 |
| SPIRE1 | 0.969093 | 0.723547 | 1.297968 | 0.833207 |
| PKD2 | 1.240236 | 0.923435 | 1.66572 | 0.152526 |
| ANKRD17 | 0.942707 | 0.71234 | 1.247573 | 0.679829 |
| UBXN4 | 0.778947 | 0.603884 | 1.00476 | 0.054429 |
| CNOT1 | 0.854896 | 0.666542 | 1.096475 | 0.216961 |
| OSBPL8 | 1.186572 | 0.925149 | 1.521867 | 0.177901 |
| TTC37 | 0.882033 | 0.673669 | 1.154842 | 0.361277 |
| DDX21 | 0.857778 | 0.684033 | 1.075654 | 0.184033 |
| GNL3L | 0.945348 | 0.720223 | 1.240842 | 0.685487 |
| AFF4 | 0.741107 | 0.569329 | 0.964715 | 0.025949 |
| EPB41 | 0.879067 | 0.671716 | 1.150426 | 0.347708 |
| NDUFA11 | 1.119476 | 0.874365 | 1.433298 | 0.370717 |
| AKAP9 | 0.967696 | 0.734652 | 1.274665 | 0.815298 |
| JMJD1C | 0.881112 | 0.680618 | 1.140667 | 0.336631 |
| ITPRIPL2 | 1.120484 | 0.862079 | 1.456345 | 0.395064 |
| FKBP2 | 1.020701 | 0.795562 | 1.309553 | 0.871974 |
| GOLIM4 | 1.069322 | 0.81208 | 1.408051 | 0.633089 |
| ARFGEF2 | 0.905717 | 0.692823 | 1.18403 | 0.468847 |
| BLOC1S1 | 1.175557 | 0.919803 | 1.502424 | 0.196311 |
| GOLGA4 | 0.893108 | 0.69 | 1.156004 | 0.390485 |
| EDF1 | 1.270608 | 0.982566 | 1.64309 | 0.067869 |
| TCEB2 | 1.31482 | 1.026614 | 1.683936 | 0.030157 |
| HIF1A | 0.926898 | 0.730373 | 1.176304 | 0.532375 |
| NDUFA3 | 1.122735 | 0.879436 | 1.433344 | 0.35289 |
| ELF1 | 0.855452 | 0.678119 | 1.079159 | 0.187765 |
| PRKDC | 1.234513 | 0.974199 | 1.564386 | 0.081225 |
| AURKAIP1 | 1.171682 | 0.913403 | 1.502994 | 0.21238 |
| NOTCH2 | 1.257895 | 0.964785 | 1.640055 | 0.090055 |
| VPS13B | 1.311815 | 1.020485 | 1.686314 | 0.034156 |
| SEC24D | 1.005674 | 0.777048 | 1.301566 | 0.965704 |
| SPTBN1 | 1.032517 | 0.806416 | 1.322012 | 0.799681 |
| C9orf16 | 1.184825 | 0.927923 | 1.512853 | 0.173812 |
| ITGAV | 1.060303 | 0.838322 | 1.341064 | 0.625156 |
| DPM3 | 1.151849 | 0.917499 | 1.446056 | 0.223198 |
| ATP11B | 1.035474 | 0.82102 | 1.305944 | 0.768445 |
| DCXR | 1.004569 | 0.827607 | 1.21937 | 0.963224 |
| NRIP1 | 0.993696 | 0.783115 | 1.260903 | 0.958494 |
| SCAND1 | 1.183326 | 0.954563 | 1.466911 | 0.124607 |
| TMEM123 | 0.900754 | 0.705587 | 1.149906 | 0.401527 |
| LAMC1 | 1.188101 | 0.933194 | 1.512638 | 0.161869 |
| CRIM1 | 1.106872 | 0.86629 | 1.414268 | 0.416767 |
| MRPL41 | 1.212149 | 0.988862 | 1.485855 | 0.064005 |
| ZNF217 | 0.968785 | 0.795895 | 1.179232 | 0.751858 |
| RPL13 | 1.06579 | 0.84898 | 1.337969 | 0.582945 |
| C12orf57 | 1.133541 | 0.901836 | 1.424777 | 0.282658 |
| RPS15 | 1.222579 | 0.973919 | 1.534728 | 0.083242 |
| LGR4 | 0.957596 | 0.756325 | 1.21243 | 0.718913 |
| MZT2B | 1.212268 | 0.980905 | 1.498201 | 0.074826 |
| GAMT | 1.046307 | 0.843124 | 1.298454 | 0.681133 |
| ETS1 | 1.100231 | 0.881019 | 1.373986 | 0.399469 |
| HERC3 | 1.299489 | 1.028523 | 1.641842 | 0.028115 |
| STAT1 | 1.112684 | 0.907588 | 1.364127 | 0.304342 |
| C19orf60 | 1.300891 | 1.051332 | 1.609689 | 0.015495 |
| MRPL12 | 1.192903 | 0.964943 | 1.474716 | 0.103068 |
| ARRDC3 | 0.863886 | 0.705145 | 1.058363 | 0.157831 |
| ITPR3 | 1.004929 | 0.825439 | 1.223448 | 0.960939 |
| CLIC4 | 0.863931 | 0.700218 | 1.065921 | 0.172432 |
| FAM198B | 1.000621 | 0.817676 | 1.224496 | 0.995195 |
| MAN1A1 | 0.985957 | 0.794203 | 1.224009 | 0.898019 |
| ANTXR1 | 1.381717 | 1.140167 | 1.67444 | 0.000974 |
| TGFBR2 | 1.097279 | 0.885628 | 1.359513 | 0.395839 |
| F2R | 1.350973 | 1.119597 | 1.630165 | 0.001698 |
| CNFN | 0.955616 | 0.781498 | 1.168527 | 0.658216 |
| AIDA | 1.351274 | 1.12222 | 1.627078 | 0.001489 |
| SERINC5 | 0.849575 | 0.70927 | 1.017634 | 0.076702 |
| FXYD3 | 0.866238 | 0.725887 | 1.033726 | 0.111345 |
| NBL1 | 0.962894 | 0.827538 | 1.120389 | 0.624688 |
| NUPR1 | 1.12806 | 0.923894 | 1.377343 | 0.236849 |
| SYCE1L | 1.117937 | 0.957591 | 1.305132 | 0.158138 |
| KIAA1244 | 1.041767 | 0.862443 | 1.258377 | 0.671167 |
| PTPRC | 1.200468 | 0.985102 | 1.462918 | 0.070114 |
| OAS3 | 1.500102 | 1.233331 | 1.824575 | 4.93E-05 |
| SLC12A2 | 0.890929 | 0.739439 | 1.073455 | 0.224543 |
| H2AFJ | 1.05148 | 0.889088 | 1.243532 | 0.557548 |
| ATP11A | 1.037716 | 0.87183 | 1.235164 | 0.676984 |
| FN1 | 1.244822 | 1.090412 | 1.421097 | 0.001191 |
| MESP1 | 1.067566 | 0.943529 | 1.207908 | 0.299488 |
| GUCY1A3 | 0.902035 | 0.753398 | 1.079997 | 0.261747 |
| COL15A1 | 1.600562 | 1.30902 | 1.957036 | 4.55E-06 |
| SULF1 | 1.359771 | 1.148824 | 1.609451 | 0.000353 |
| EPHA3 | 1.039099 | 0.868492 | 1.243219 | 0.675119 |
| GADD45G | 1.061935 | 0.921154 | 1.224232 | 0.407588 |
| LRRC26 | 0.992401 | 0.87896 | 1.120482 | 0.901973 |
| COL3A1 | 1.627415 | 1.394105 | 1.899772 | 6.90E-10 |
| COX7A1 | 1.010271 | 0.860791 | 1.185708 | 0.90046 |
| HSPB1 | 1.033725 | 0.887264 | 1.204361 | 0.670469 |
| MYO6 | 1.032339 | 0.883415 | 1.206367 | 0.688852 |
| THBS2 | 1.535213 | 1.325367 | 1.778285 | 1.09E-08 |
| RAMP1 | 1.130048 | 0.98058 | 1.302299 | 0.091214 |
| CXCL9 | 1.040406 | 0.907664 | 1.192562 | 0.569494 |
| CDC42EP5 | 0.873949 | 0.774646 | 0.985983 | 0.028572 |
| LYZ | 1.213077 | 1.072708 | 1.371814 | 0.00208 |
| NUDT8 | 0.961612 | 0.847492 | 1.0911 | 0.543652 |
| CXCL10 | 1.070951 | 0.932119 | 1.23046 | 0.33322 |
| PCSK1N | 1.272296 | 1.119953 | 1.445362 | 0.000215 |
| LRRN1 | 1.115402 | 0.992927 | 1.252984 | 0.065715 |
| GNMT | 0.994029 | 0.88527 | 1.11615 | 0.919317 |
| TPM2 | 0.937737 | 0.826597 | 1.063819 | 0.317899 |
| F5 | 1.114247 | 0.995142 | 1.247608 | 0.060719 |
| AZGP1 | 0.933484 | 0.849467 | 1.025809 | 0.152598 |
