# Supplementary figures and images for: Identification of cuproptosis -related subtypes, the development of a prognosis model, and characterization of tumor microenvironment infiltration in prostate cancer
Source: Front Immunol. 2022 Sep 20;13:974034. doi: 10.3389/fimmu.2022.974034 (PMC9530990; doi:10.3389/fimmu.2022.974034)

# Correlation between expression and immune infiltrates in PRAD

Symbol

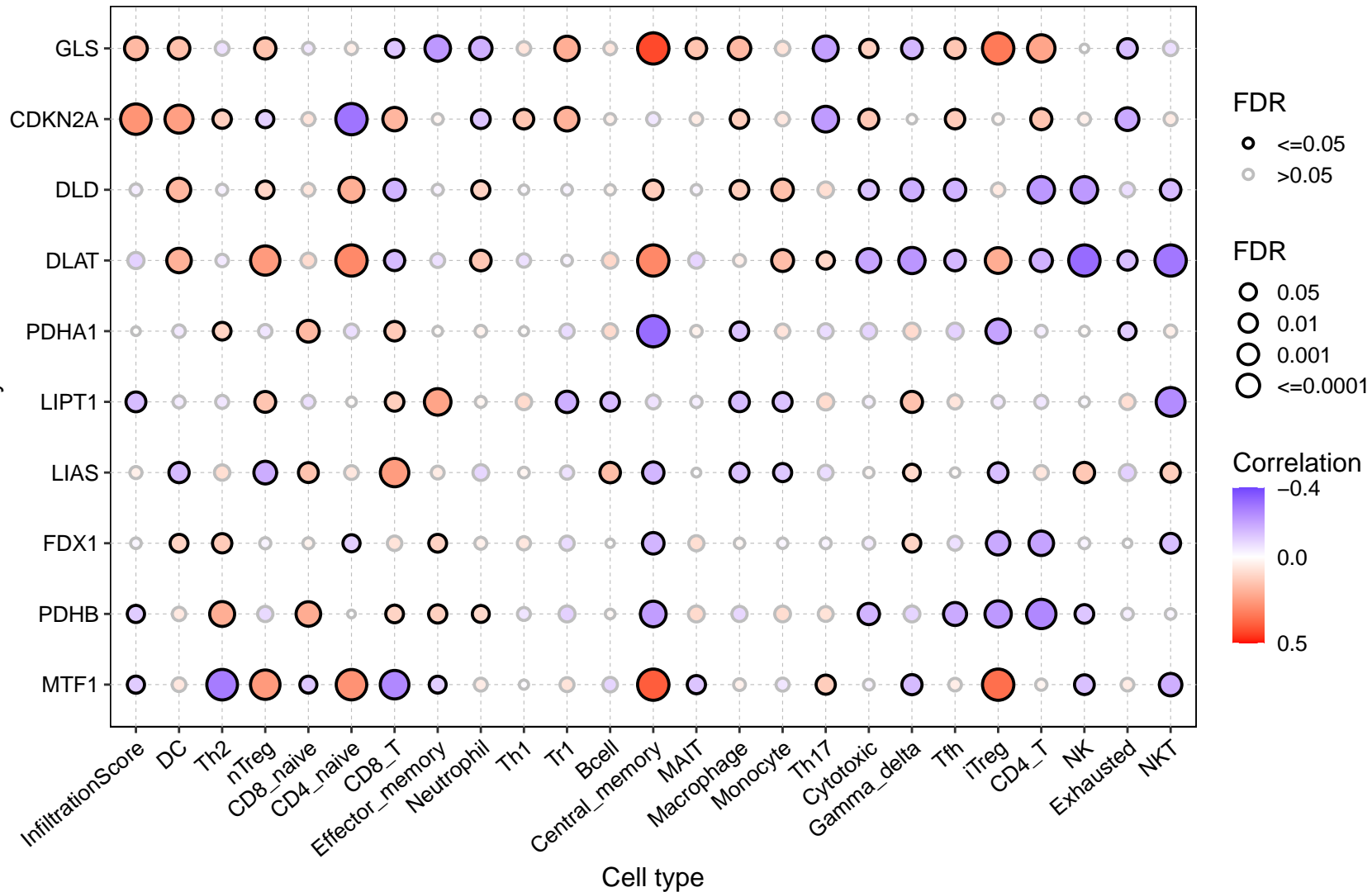

Supplement: Supplementary file 1 [file DataSheet_1.zip › supplementary materials/Figure S1.pdf]

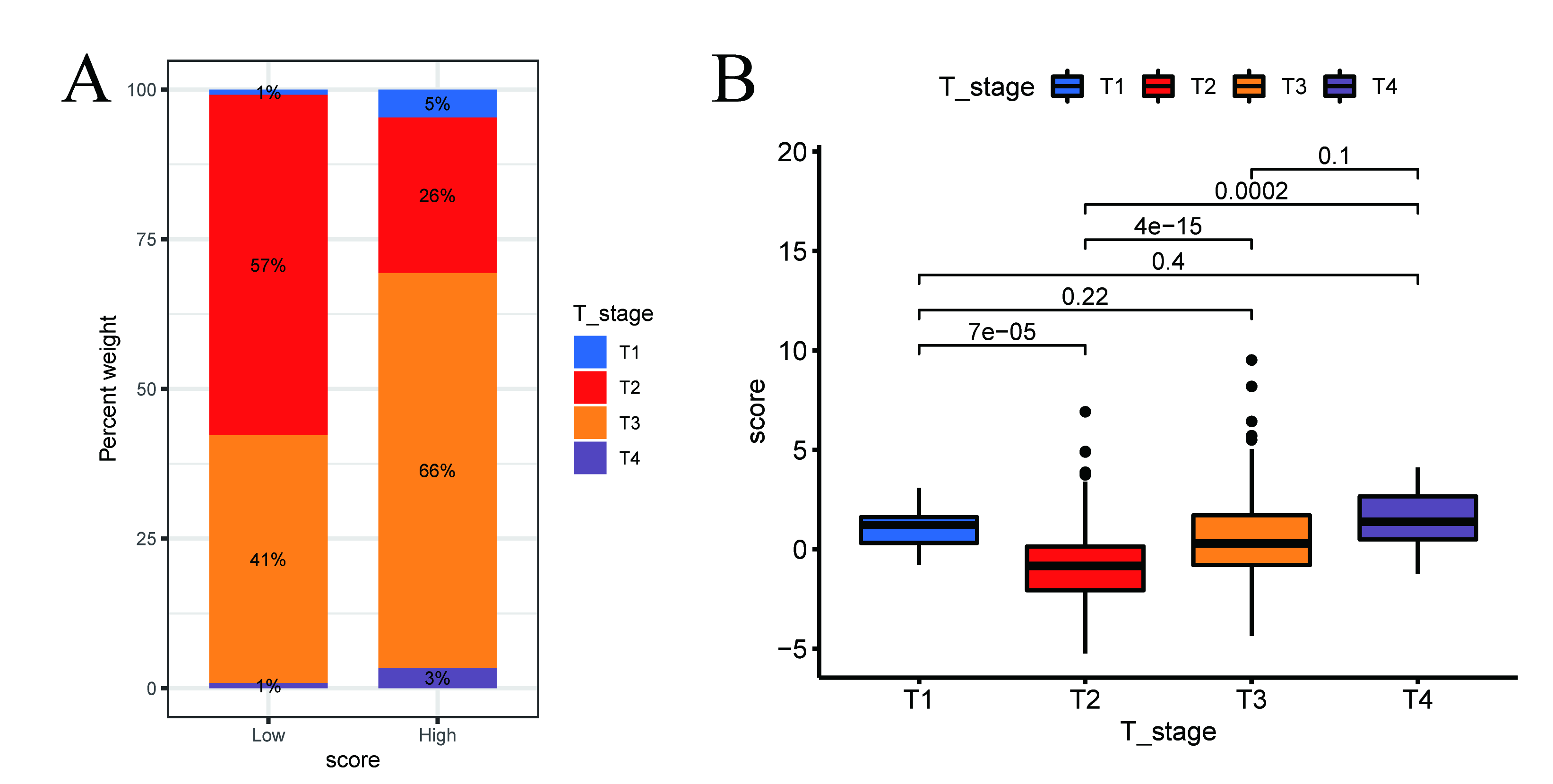

Supplement: Supplementary file 1 [file DataSheet_1.zip › supplementary materials/Figure S2.tif]
